# 5-(4-Hy­droxy­benzyl­idene)-2,2-dimethyl-1,3-dioxane-4,6-dione

**DOI:** 10.1107/S1600536810032149

**Published:** 2010-08-18

**Authors:** Wu-Lan Zeng

**Affiliations:** a MicroScale Science Institute, Department of Chemistry and Chemical Engineering, Weifang University, Weifang 261061, People’s Republic of China

## Abstract

The title compound, C_13_H_12_O_5_, was prepared by the reaction of 2,2-dimethyl-1,3-dioxane-4,6-dione and 4-hy­droxy­benz­alde­hyde in ethanol. The 1,3-dioxane ring is in a distorted boat conformation. In the crystal, inversion dimers linked by pairs of O—H⋯O hydrogen bonds generate *R*_2_^2^(20) rings.

## Related literature

For a related structure, see: Zeng & Jian (2009 ▶).
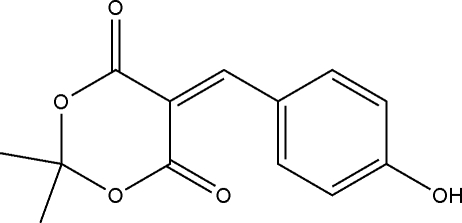


## Experimental

### 

#### Crystal data

C_13_H_12_O_5_
*M* *_r_* = 248.23
Monoclinic, 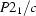

*a* = 13.900 (3) Å
*b* = 10.249 (2) Å
*c* = 8.1357 (16) Å
β = 94.47 (3)°
*V* = 1155.5 (4) Å^3^
*Z* = 4
Mo *K*α radiation
μ = 0.11 mm^−1^
*T* = 293 K
0.20 × 0.16 × 0.11 mm

#### Data collection

Bruker SMART CCD diffractometer
10923 measured reflections
2644 independent reflections
2402 reflections with *I* > 2σ(*I*)
*R*_int_ = 0.035

#### Refinement

*R*[*F* ^2^ > 2σ(*F* ^2^)] = 0.044
*wR*(*F* ^2^) = 0.116
*S* = 1.05
2644 reflections
163 parameters
H-atom parameters constrained
Δρ_max_ = 0.25 e Å^−3^
Δρ_min_ = −0.28 e Å^−3^

### 

Data collection: *SMART* (Bruker, 1997 ▶); cell refinement: *SAINT* (Bruker, 1997 ▶); data reduction: *SAINT*; program(s) used to solve structure: *SHELXS97* (Sheldrick, 2008 ▶); program(s) used to refine structure: *SHELXL97* (Sheldrick, 2008 ▶); molecular graphics: *SHELXTL* (Sheldrick, 2008 ▶); software used to prepare material for publication: *SHELXTL*.

## Supplementary Material

Crystal structure: contains datablocks global, I. DOI: 10.1107/S1600536810032149/hb5605sup1.cif

Structure factors: contains datablocks I. DOI: 10.1107/S1600536810032149/hb5605Isup2.hkl

Additional supplementary materials:  crystallographic information; 3D view; checkCIF report

## Figures and Tables

**Table 1:** Hydrogen-bond geometry (Å, °)

*D*—H⋯*A*	*D*—H	H⋯*A*	*D*⋯*A*	*D*—H⋯*A*
O5—H5*A*⋯O2^i^	0.82	1.98	2.7919 (14)	170

Symmetry code: (i) 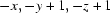.

**Fig. 1.**
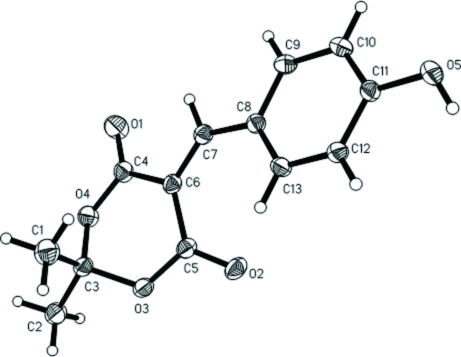
The molecular structure of (I), drawn with 30% probability ellipsoids and spheres of arbritrary size for the H atoms.

## supplementary crystallographic information

### Comment

We have recently reported the crystal structure of 5-(2-fluorobenzylidene)-2,2-dimethyl-1,3-dioxane-4,6-dione (Zeng & Jian 2009). As part of our search for new Meldrum's acid derivatives, the title compound, (I) (Fig. 1), has been synthesized and its structure is reported here. The crystal structure analysis confirms the title compound with atom C7 bridged by the 1,3-dioxane ring via C7═C6 double bond [1.3580 (16)Å] and the phenyl ring via C7-C8 single bond [1.4513 (15)Å], forming the C6-C7-C8 bond angle of 134.33 (10)°. The crystal structure is stabilized by weak intermolecular O—H···O hydrogen bonds (Table 1).

### Experimental

A mixture of malonic acid (6.24 g, 0.06 mol) and acetic anhydride (9 ml) in strong sulfuric acid (0.25 ml) was stirred with water at 303 K, After dissolving, propan-2-one (3.48 g, 0.06 mol) was added dropwise into solution for 1 h. The reaction was allowed to proceed for 2 h. The mixture was cooled and filtered, and then an ethanol solution of 4-hydroxybenzaldehyde (7.32g, 0.06 mol) was added. The solution was then filtered and concentrated. Yellow blocks of (I) were obtained by evaporation of an petroleum ether-acetone (2:1 *v*/*v*) solution of the title compound at room temperature over a period of several days.

### Refinement

The H atoms were placed in calculated positions (C—H = 0.93–0.97 Å), and refined as riding with *U*_iso_(H) = 1.2*U*_eq_(C) or 1.5U_eq_(methyl C).

### Crystal data

**Table:**

C_13_H_12_O_5_	*F*(000) = 520
*M**_r_* = 248.23	*D*_x_ = 1.427 Mg m^−^^3^
Monoclinic, *P*2_1_/*c*	Mo *K*α radiation, λ = 0.71073 Å
Hall symbol: -P 2ybc	Cell parameters from 2644 reflections
*a* = 13.900 (3) Å	θ = 3.2–27.5°
*b* = 10.249 (2) Å	µ = 0.11 mm^−^^1^
*c* = 8.1357 (16) Å	*T* = 293 K
β = 94.47 (3)°	Block, yellow
*V* = 1155.5 (4) Å^3^	0.20 × 0.16 × 0.11 mm
*Z* = 4	

### Data collection

**Table:**

Bruker SMART CCD diffractometer	2402 reflections with *I* > 2σ(*I*)
Radiation source: fine-focus sealed tube	*R*_int_ = 0.035
graphite	θ_max_ = 27.5°, θ_min_ = 3.2°
phi and ω scans	*h* = −17→18
10923 measured reflections	*k* = −13→13
2644 independent reflections	*l* = −10→10

### Refinement

**Table:**

Refinement on *F*^2^	Primary atom site location: structure-invariant direct methods
Least-squares matrix: full	Secondary atom site location: difference Fourier map
*R*[*F*^2^ > 2σ(*F*^2^)] = 0.044	Hydrogen site location: inferred from neighbouring sites
*wR*(*F*^2^) = 0.116	H-atom parameters constrained
*S* = 1.05	*w* = 1/[σ^2^(*F*_o_^2^) + (0.0685*P*)^2^ + 0.2332*P*] where *P* = (*F*_o_^2^ + 2*F*_c_^2^)/3
2644 reflections	(Δ/σ)_max_ < 0.001
163 parameters	Δρ_max_ = 0.25 e Å^−^^3^
0 restraints	Δρ_min_ = −0.28 e Å^−^^3^

### Special details

**Table:**

Geometry. All e.s.d.'s (except the e.s.d. in the dihedral angle between two l.s. planes) are estimated using the full covariance matrix. The cell e.s.d.'s are taken into account individually in the estimation of e.s.d.'s in distances, angles and torsion angles; correlations between e.s.d.'s in cell parameters are only used when they are defined by crystal symmetry. An approximate (isotropic) treatment of cell e.s.d.'s is used for estimating e.s.d.'s involving l.s. planes.
Refinement. Refinement of *F*^2^ against ALL reflections. The weighted *R*-factor *wR* and goodness of fit *S* are based on *F*^2^, conventional *R*-factors *R* are based on *F*, with *F* set to zero for negative *F*^2^. The threshold expression of *F*^2^ > σ(*F*^2^) is used only for calculating *R*-factors(gt) *etc*. and is not relevant to the choice of reflections for refinement. *R*-factors based on *F*^2^ are statistically about twice as large as those based on *F*, and *R*- factors based on ALL data will be even larger.

### Fractional atomic coordinates and isotropic or equivalent isotropic displacement parameters (Å^2^)

**Table:**

	*x*	*y*	*z*	*U*_iso_*/*U*_eq_	
O4	0.41524 (6)	0.57554 (9)	0.12495 (11)	0.0401 (2)	
O3	0.35540 (6)	0.47383 (10)	0.35423 (10)	0.0409 (2)	
O2	0.21646 (6)	0.52747 (11)	0.44151 (11)	0.0468 (3)	
O5	−0.22815 (6)	0.59477 (10)	0.25091 (12)	0.0468 (3)	
H5A	−0.2323	0.5600	0.3408	0.070*	
C7	0.15802 (8)	0.63304 (11)	0.10210 (14)	0.0317 (2)	
H7A	0.1617	0.6830	0.0074	0.038*	
C11	−0.13455 (8)	0.59747 (11)	0.21578 (14)	0.0316 (2)	
C8	0.05963 (8)	0.61437 (10)	0.14540 (13)	0.0288 (2)	
C13	0.02894 (8)	0.52087 (11)	0.25578 (14)	0.0313 (2)	
H13A	0.0739	0.4638	0.3070	0.038*	
C12	−0.06604 (8)	0.51181 (11)	0.28980 (14)	0.0320 (3)	
H12A	−0.0848	0.4483	0.3625	0.038*	
O1	0.32758 (7)	0.71114 (11)	−0.03290 (14)	0.0580 (3)	
C5	0.26819 (8)	0.52924 (12)	0.32898 (14)	0.0337 (3)	
C6	0.24587 (8)	0.59391 (11)	0.16895 (14)	0.0318 (2)	
C9	−0.01161 (8)	0.69363 (11)	0.06446 (14)	0.0337 (3)	
H9A	0.0060	0.7520	−0.0155	0.040*	
C10	−0.10703 (8)	0.68732 (11)	0.10023 (15)	0.0355 (3)	
H10A	−0.1525	0.7428	0.0474	0.043*	
C4	0.32958 (9)	0.63232 (12)	0.07624 (15)	0.0373 (3)	
C3	0.41508 (8)	0.45675 (13)	0.21746 (14)	0.0366 (3)	
C2	0.51631 (10)	0.43795 (19)	0.29247 (18)	0.0544 (4)	
H2A	0.5351	0.5122	0.3594	0.082*	
H2B	0.5593	0.4289	0.2064	0.082*	
H2C	0.5192	0.3608	0.3595	0.082*	
C1	0.37811 (12)	0.34509 (14)	0.11015 (19)	0.0525 (4)	
H1A	0.3133	0.3633	0.0669	0.079*	
H1B	0.3789	0.2665	0.1744	0.079*	
H1C	0.4186	0.3343	0.0207	0.079*	

### Atomic displacement parameters (Å^2^)

**Table:**

	*U*^11^	*U*^22^	*U*^33^	*U*^12^	*U*^13^	*U*^23^
O4	0.0294 (4)	0.0477 (5)	0.0443 (5)	−0.0016 (3)	0.0091 (3)	0.0076 (4)
O3	0.0317 (4)	0.0625 (6)	0.0287 (4)	0.0045 (4)	0.0036 (3)	0.0062 (4)
O2	0.0318 (4)	0.0783 (7)	0.0309 (4)	−0.0035 (4)	0.0067 (3)	0.0073 (4)
O5	0.0273 (4)	0.0666 (6)	0.0468 (5)	0.0088 (4)	0.0051 (4)	0.0100 (5)
C7	0.0339 (6)	0.0314 (5)	0.0302 (5)	−0.0014 (4)	0.0052 (4)	0.0013 (4)
C11	0.0270 (5)	0.0372 (6)	0.0303 (5)	0.0024 (4)	0.0003 (4)	−0.0045 (4)
C8	0.0290 (5)	0.0293 (5)	0.0281 (5)	−0.0001 (4)	0.0015 (4)	−0.0026 (4)
C13	0.0286 (5)	0.0316 (5)	0.0334 (5)	0.0038 (4)	0.0007 (4)	0.0037 (4)
C12	0.0310 (5)	0.0352 (5)	0.0299 (5)	0.0005 (4)	0.0026 (4)	0.0039 (4)
O1	0.0496 (6)	0.0612 (6)	0.0660 (7)	0.0073 (5)	0.0224 (5)	0.0309 (5)
C5	0.0273 (5)	0.0443 (6)	0.0294 (5)	−0.0052 (4)	0.0025 (4)	0.0005 (4)
C6	0.0298 (5)	0.0360 (5)	0.0301 (5)	−0.0023 (4)	0.0063 (4)	0.0010 (4)
C9	0.0365 (6)	0.0311 (5)	0.0327 (5)	−0.0015 (4)	−0.0015 (4)	0.0038 (4)
C10	0.0334 (6)	0.0347 (6)	0.0373 (6)	0.0052 (4)	−0.0047 (4)	0.0032 (5)
C4	0.0332 (6)	0.0393 (6)	0.0406 (6)	−0.0009 (4)	0.0100 (5)	0.0050 (5)
C3	0.0326 (6)	0.0481 (7)	0.0297 (5)	0.0031 (5)	0.0051 (4)	0.0032 (5)
C2	0.0337 (6)	0.0894 (11)	0.0402 (7)	0.0138 (7)	0.0031 (5)	0.0039 (7)
C1	0.0594 (9)	0.0470 (8)	0.0511 (8)	−0.0002 (6)	0.0036 (7)	−0.0049 (6)

### Geometric parameters (Å, °)

**Table:**

O4—C4	1.3564 (15)	C12—H12A	0.9300
O4—C3	1.4314 (15)	O1—C4	1.1991 (15)
O3—C5	1.3399 (15)	C5—C6	1.4722 (16)
O3—C3	1.4493 (14)	C6—C4	1.4882 (16)
O2—C5	1.2076 (15)	C9—C10	1.3814 (17)
O5—C11	1.3539 (14)	C9—H9A	0.9300
O5—H5A	0.8200	C10—H10A	0.9300
C7—C6	1.3580 (16)	C3—C2	1.5019 (17)
C7—C8	1.4513 (15)	C3—C1	1.5053 (19)
C7—H7A	0.9300	C2—H2A	0.9600
C11—C10	1.3907 (17)	C2—H2B	0.9600
C11—C12	1.3968 (16)	C2—H2C	0.9600
C8—C13	1.4023 (15)	C1—H1A	0.9600
C8—C9	1.4044 (15)	C1—H1B	0.9600
C13—C12	1.3730 (16)	C1—H1C	0.9600
C13—H13A	0.9300		
			
C4—O4—C3	118.76 (9)	C8—C9—H9A	119.1
C5—O3—C3	119.91 (9)	C9—C10—C11	119.52 (10)
C11—O5—H5A	109.5	C9—C10—H10A	120.2
C6—C7—C8	134.33 (10)	C11—C10—H10A	120.2
C6—C7—H7A	112.8	O1—C4—O4	118.33 (11)
C8—C7—H7A	112.8	O1—C4—C6	125.42 (12)
O5—C11—C10	118.41 (10)	O4—C4—C6	116.21 (10)
O5—C11—C12	122.02 (11)	O4—C3—O3	108.99 (10)
C10—C11—C12	119.56 (10)	O4—C3—C2	106.43 (11)
C13—C8—C9	117.19 (10)	O3—C3—C2	106.12 (10)
C13—C8—C7	125.81 (10)	O4—C3—C1	110.86 (10)
C9—C8—C7	116.95 (10)	O3—C3—C1	110.31 (11)
C12—C13—C8	121.38 (10)	C2—C3—C1	113.88 (13)
C12—C13—H13A	119.3	C3—C2—H2A	109.5
C8—C13—H13A	119.3	C3—C2—H2B	109.5
C13—C12—C11	120.30 (10)	H2A—C2—H2B	109.5
C13—C12—H12A	119.9	C3—C2—H2C	109.5
C11—C12—H12A	119.9	H2A—C2—H2C	109.5
O2—C5—O3	117.55 (11)	H2B—C2—H2C	109.5
O2—C5—C6	125.47 (11)	C3—C1—H1A	109.5
O3—C5—C6	116.88 (10)	C3—C1—H1B	109.5
C7—C6—C5	127.45 (10)	H1A—C1—H1B	109.5
C7—C6—C4	115.66 (10)	C3—C1—H1C	109.5
C5—C6—C4	116.62 (10)	H1A—C1—H1C	109.5
C10—C9—C8	121.84 (10)	H1B—C1—H1C	109.5
C10—C9—H9A	119.1		

### Hydrogen-bond geometry (Å, °)

**Table:**

*D*—H···*A*	*D*—H	H···*A*	*D*···*A*	*D*—H···*A*
O5—H5A···O2^i^	0.82	1.98	2.7919 (14)	170

Symmetry codes: (i) −*x*, −*y*+1, −*z*+1.
